# Falls Prevention Among Older Adults in Rural Communities: Protocol for a Scoping Review

**DOI:** 10.2196/63716

**Published:** 2025-07-21

**Authors:** Megan Funk, Juanita-Dawne Bacsu, Melba Sheila D’Souza, Anila Virani, Zahra Rahemi, Matthew Lee Smith

**Affiliations:** 1 Population Health and Aging Rural Research Centre School of Nursing Thompson Rivers University Kamloops, BC Canada; 2 School of Nursing Clemson University Clemson, SC United States; 3 Department of Health Behavior School of Public Health Texas A&M University College Station, TX United States

**Keywords:** falls prevention, rural, older adults, aging, perceptions, protocol, scoping review

## Abstract

**Background:**

Falls are a critical source of injury and hospitalization and leave many older adults unable to return home, especially in rural communities with limited access to health care and support services. Studying falls prevention among rural older adults is essential because they may face an increased risk of falls due to unique environmental factors, geography, and outdoor activities. Moreover, rural older adults may have limited awareness regarding fall-related risks and preventive activities.

**Objective:**

The objective of this scoping review is to explore the literature about falls prevention from the perspectives of older adults living within a rural context. This review protocol aims to identify the search parameters and methodology that will be used in the scoping review.

**Methods:**

This scoping review will be guided by Arksey and O’Malley’s 5-step methodological framework. We will search for relevant peer-reviewed English language literature from 5 databases: CINAHL, PubMed, Academic Search Complete, PsycINFO, and Scopus. The reference lists of relevant studies will be hand-searched to identify papers. Inclusion criteria (English language, peer-reviewed journal papers, original research, focusing on rural perspectives to support falls prevention, and published from January 2013 to December 31, 2023) will be used to determine the eligibility of the journal papers. The data from the included papers will be extracted using a standardized table and analyzed using thematic analysis.

**Results:**

This protocol was registered with the Open Science Framework on June 26, 2024. The scoping review’s data collection and analysis were conducted from September to December 2024. Results from the review will be distributed through publication in a peer-reviewed journal paper, conference presentation, webinar, and a rural community workshop.

**Conclusions:**

Understanding rural older adults’ perspectives of falls prevention is critical to supporting independence and healthy aging in rural communities. This review’s findings about falls prevention may have important implications for rural community leaders, policy makers, and health practitioners working to support falls prevention in rural communities.

**International Registered Report Identifier (IRRID):**

PRR1-10.2196/63716

## Introduction

Falls are a substantial public health issue and a leading cause of mortality and morbidity among older adults worldwide [1]. The World Health Organization (WHO) reports that every year approximately 684,000 people die from falls [2]. A fall can be defined as an event in which an individual unintentionally comes to rest on the floor [3].

Compared to urban older adults, rural older adults report a higher risk of falls and experience a higher incidence of falls [4]. This increased risk of falls is due to differences in the physical environment, lifestyle activities, social issues, and geospatially dispersed health-related services and resources [5,6]. Rural older adults face unique challenges in accessing treatment for fall-related injuries due to limited public transportation, financial challenges, dispersed geography, and inadequate access to health care and support services [7].

Falls can lead to severe injury, dependence, death, and institutionalization among older adults [8,9] and are a critical public health issue due to their economic cost to the health care system [4,10]. The Government of Canada [11] reports that falls cost the health care system approximately CAD 2 billion (USD 1.48 billion) each year. Falls often result in debilitating injuries, require hospitalization, and leave many older adults unable to return home, especially for rural older adults with limited access to health care and support services [10,12]. Given the economic cost and the potentially life-threatening consequences of falls, there is an urgent need for research to address falls prevention within a rural context.

Current literature about rural older adults’ perspectives on falls prevention in rural communities is limited. Although 2 literature reviews exist on falls, they focus primarily on rural older adults in the Australian context. For example, Boehm and colleagues [13] completed a literature review focusing on falls epidemiology in rural Australia. Similarly, Peters and colleagues [14] focused on the rural Australian context and examined paramedicine practices in supporting rural falls management. However, no existing literature reviews focus on the viewpoints of rural older adults. Moreover, existing research highlights the challenges of dissemination of fall-related programming and services in rural communities [15,16].

Given that falls are largely preventable, there is a critical need to understand rural older adults’ perspectives on ways to reduce fall-related risks and injury. For example, rural older adults can provide unique insight about the contributing factors and risks to support falls prevention. By examining rural older adults’ perspectives, we can develop community-informed interventions to support falls prevention and awareness within a rural context. Accordingly, this scoping review protocol outlines the methodology that will be used to examine rural older adults’ perspectives on falls prevention. By focusing on the perspectives of rural older adults, this review’s findings will have important implications for rural policy makers, health practitioners, and community leaders working to support rural aging.

## Methods

### Study Framework

This scoping review will follow Arksey and O’Malley’s [17] scoping review framework and the PRISMA-ScR (Preferred Reporting Items for Systematic reviews and Meta-Analyses extension for Scoping Reviews) checklist (Multimedia Appendix 1) [18]. Guided by Arksey and O’Malley’s framework, this study will be organized into 5 stages: (1) identification of the research question; (2) examination of relevant studies; (3) study selection; (4) data extraction; and (5) collating, summarizing, and reporting the research findings [17].

### Step 1: Identification of the Research Question

The primary and secondary research questions were created to examine the existing literature on rural older adults’ perspectives of falls prevention. Specifically, the primary question was developed to understand how rural older adults view falls prevention overall. The secondary questions were created to explore how rural older adults view their risk of falls and to identify what they consider to be contributing factors of falls. It is important to note that these questions focus on examining rural older adults’ perspectives of prevention and risk, without focusing on fall experiences. Questions about fall experiences were avoided to align with existing research that shows that older adults focus on fall perspectives and implications rather than the fall experience itself [19]. Accordingly, it is important to emphasize that our study focuses on understanding rural older adults’ perspectives of falls prevention and not experiences. We have included our primary and secondary research questions below:

1. Primary research question: What are rural older adults’ perceptions about falls prevention?
2. Secondary research questions: (1) How do older adults living in rural communities perceive their risk of falls? (2) What do rural older adults identify as the contributing factors of falls? (3) What are rural older adults’ perspectives of mitigation strategies to reduce falls?

### Step 2: Identification of Relevant Studies

An expert librarian was consulted to recommend the most relevant databases. Based on the librarian’s expertise, a broad search on EBSCO Discovery Service was conducted to identify the most applicable databases. Based on the librarian’s expert guidance, 5 databases were searched: CINAHL, PubMed, Academic Search Complete, PsycINFO, and Scopus. Studies were also obtained by searching for bibliographic references of relevant studies. The time frame of the search will focus on papers published over the past 10 years from January 2013 to December 31, 2023. These dates were chosen to identify the most recent and up-to-date literature available. The keywords that were included in this search strategy are documented in Table 1. For example, the search string for CINAHL was (falls prevention or preventing falls or prevent falls or reduce falls or fall mitigation or fall risk reduction) AND (older adults or elderly or seniors or geriatrics or geriatric or seniors or aging or older people) AND (rural or rurality or underserved) AND (perceptions or attitudes or opinion or perspectives or view). Regarding our search terms, it is important to note that existing research shows that perspectives and experiences cannot be used interchangeably. For example, older adults tend to focus on the antecedents and consequences of falls rather than the fall experience itself [19]. Consequently, our search terms focused strategically on words related to prevention rather than experiences because our research aims to highlight the perspectives of rural older adults.

**Table 1:** Keyword search strategy.^a^

Concept	Keywords
Falls prevention	Preventing falls, prevent falls, reduce falls, fall mitigation, fall risk reduction
Older adults	Elderly, seniors, geriatric, geriatrics, aging, older people
Rural	Rural, rurality, underserved
Perceptions	Attitudes, opinion, perspectives, view

^a^Databases used were CINAHL, PubMed, Academic Search Complete, PsycINFO, and Scopus.

### Inclusion and Exclusion Criteria

Publications will be included in this scoping review if they meet the following 4 criteria: (1) full text, peer-reviewed journal papers; (2) written in the English language; (3) published from 2013 to 2024; and (4) focus on older adults’ perceptions about falls among those living in rural communities. This study will include publications with different research methods such as qualitative, quantitative, and mixed methods studies. Exclusion criteria will include the following: (1) papers that do not report on original research (such as commentaries and editorials), (2) papers published in languages that are not English, and (3) papers that do not address the research topic.

### Step 3: Study Selection

Five electronic databases (CINAHL, PubMed, Academic Search Complete, PsycINFO, and Scopus) will be searched for relevant literature, and the results will be imported into Covidence software to aid in the organization and management in the review’s selection and screening process [20]. Guided by the inclusion and exclusion criteria, 2 reviewers will perform the study selection process. A PRISMA diagram will be used to document the scoping review’s study identification, selection, and screening process.

### Step 4: Charting the Data

The data will be charted and extracted using a standardized data extraction table in Microsoft Word. The table will consist of different categories to help chart and organize the data, including the author, publication year, country of origin, purpose of the study, methods, and findings. We will also have a category to collect information to address the question-related themes, including information related to fall-related risks, contributing factors of falls, and/or mitigation strategies. Exploring this information will allow the researchers to effectively synthesize the existing information on falls prevention among rural older adults. This table will be assessed with 4 papers to ensure that the table adequately captures the data, and no missing categories are required to address the research questions.

### Step 5: Collating, Summarizing, and Reporting the Research Findings

Guided by Braun and Clarke [21], we will analyze our scoping review data by using thematic analysis. Specifically, our process of thematic analysis will consist of using a deductive approach where our research process will be guided by our pre-established research questions. For examples, our codes will be guided by using our research questions (fall-related risks, contributing factors, and mitigation strategies), and our themes will be created by organizing our codes into theme and subtheme piles to ensure that our themes align with addressing our research objectives. Our theme names will be refined to ensure clarity, uniqueness, and that they provide insight into addressing each of our research questions.

Measures will be taken to support rigor in the data. For example, dependability will be accomplished by keeping a comprehensive audit trail to document the research steps and decisions made throughout the process [21]. Reflexivity will be integrated by reflecting and mitigating authors’ potential biases and assumptions during the data synthesis and the development of themes. Additionally, confirmability will be incorporated through peer debriefing and reviewing interpretations to ensure the findings are representative of the data.

## Results

This protocol was registered with the Open Science Framework on June 26, 2024. The scoping review’s data collection and analysis were conducted from September to December 2024. Results from this review will be distributed through publication in a peer-reviewed journal paper, conference presentation, webinar, and a rural community workshop.

## Discussion

### Overview

Our scoping review will aim to examine the literature on rural older adults’ perspectives of falls prevention. Supporting falls prevention and independence of older adults in rural communities is critical for several unique factors ranging from education to geography. More specifically, research suggests that rural older adults often lack education and awareness of falls prevention and have an increased risk of falling compared to their urban counterparts [4]. Research shows that increased risk of falls may be related to rural older adults’ day-to-day activities, geography [5], and physical environments such as uneven walkways [22]. Across diverse global contexts, rural older adults often face limited treatment options due to issues of inadequate public transportation, geographical distance, social isolation, and reduced access to health care and support services [4,23-26]. Consequently, falls prevention is vital to supporting older adults’ independence to protect themselves from issues of social isolation, loss of selfhood, and fears of dependency [27]. Additionally, falls can cause severe injury, dependence, and mortality among older adults and are costly to the health care system [2]. Each year, falls cost the health care system in Canada approximately CAD 2 billion (USD 1.48 billion) [7] and upwards of CAD 68 billion (~USD 50 billion) worldwide [26].

Although research addresses falls prevention among older adults, there is a notable lack of discussion focusing directly on the perspectives of rural older adults. However, older adults’ perspectives of falls prevention are important because research shows that they have unique views regarding falls [19,28]. For example, Zecevic and colleagues [19] reported that older adults tend to focus on the causes and implications of falls, whereas researchers often emphasize the fall itself. Accordingly, the findings from this review will have important implications for rural policy makers, practitioners, and community leaders who are dedicated to addressing falls prevention in rural communities. Understanding the unique views and perspectives of older adults will inform the development of targeted strategies and actions to enhance falls prevention in rural communities.

### Limitations

This scoping review will be completed in a rigorous and comprehensive manner. However, it is important to note that our review may include potential limitations. For example, our review will be limited to literature published in peer-reviewed journal papers in the English language. Thus, information in gray literature will be excluded from this study. Future reviews may expand their search to include gray literature and unpublished materials. It should be noted that future reviews using gray literature should be cautious, as gray literature is often not peer-reviewed. Another limitation is that only papers in the English language will be included in this review. However, there may be relevant peer-reviewed literature published in languages other than English. Accordingly, future research should consider examining non-English language literature on rural older adults’ perspectives of falls prevention.

### Conclusion

Understanding rural older adults’ perspectives of falls prevention is essential to supporting independence and healthy aging in rural communities. Although research addresses falls prevention among older adults, there is a notable lack of discussion focusing directly on the perspectives of rural older adults. This scoping review on falls prevention may have important implications for rural community leaders, policy makers, and health practitioners working to support falls prevention for older adults living in rural communities.

## Appendix

Multimedia Appendix 1 PRISMA-ScR (Preferred Reporting Items for Systematic reviews and Meta-Analyses extension for Scoping Reviews) checklist.

## Abbreviations

PRISMA-ScR: Preferred Reporting Items for Systematic reviews and Meta-Analyses extension for Scoping Reviews
WHO: World Health Organization

## Data Availability

The data collected during the scoping review will be available on the Open Science Framework repository and as supplementary files with our scoping review manuscript.

## References

[1] Yang Y, Ye Q, Yao M, Yang Y, Lin T (2022). Development of the Home-Based Fall Prevention Knowledge (HFPK) questionnaire to assess home-based fall prevention knowledge levels among older adults in China. BMC Public Health.

[2] Step safely: strategies for preventing and managing falls across the life-course. World Health Organization.

[3] Alshammari SA, Alhassan AM, Aldawsari MA, Bazuhair FO, Alotaibi FK, Aldakhil AA, Abdulfattah FW (2018). Falls among elderly and its relation with their health problems and surrounding environmental factors in Riyadh. J Family Community Med.

[4] Zhang L, Ding Z, Qiu L, Li A (2019). Falls and risk factors of falls for urban and rural community-dwelling older adults in China. BMC Geriatr.

[5] Towne S, Probst J, Smith M, Salinas M, Ory M (2016). Rural health and aging: global perspectives. Encyclopedia of Geropsychology.

[6] Weber V, White A, McIlvried R (2008). An electronic medical record (EMR)-based intervention to reduce polypharmacy and falls in an ambulatory rural elderly population. J Gen Intern Med.

[7] Age-friendly communities. Government of Canada.

[8] Hosseinpour H, El-Qawaqzeh K, Stewart C, Akl MN, Anand T, Culbert MH, Nelson A, Bhogadi SK, Joseph B (2022). Emergency readmissions following geriatric ground-level falls: How does frailty factor in?. Injury.

[9] Granbom M, Clemson L, Roberts L, Hladek MD, Okoye SM, Liu M, Felix C, Roth DL, Gitlin LN, Szanton S (2019). Preventing falls among older fallers: study protocol for a two-phase pilot study of the multicomponent LIVE LiFE program. Trials.

[10] Florence CS, Bergen G, Atherly A, Burns E, Stevens J, Drake C (2018). Medical costs of fatal and nonfatal falls in older adults. J Am Geriatr Soc.

[11] Seniors' Falls in Canada - Infographic. Government of Canada.

[12] Ambrose AF, Paul G, Hausdorff JM (2013). Risk factors for falls among older adults: a review of the literature. Maturitas.

[13] Boehm J, Franklin RC, King JC (2014). Falls in rural and remote community dwelling older adults: a review of the literature. Aust J Rural Health.

[14] Peters A, Lim D, Naidoo N (2023). Down with falls! Paramedicine scope regarding falls amongst older adults in rural and remote communities: a scoping review. Aust J Rural Health.

[15] Smith M, Towne S, Pickett A, Horel S, Kulinski K, Kaye LW (2021). Evidence-based programs. Handbook of Rural Aging.

[16] Smith ML, Towne SD, Herrera-Venson A, Cameron K, Horel SA, Ory MG, Gilchrist CL, Schneider EC, DiCocco C, Skowronski S (2018). Delivery of fall prevention interventions for at-risk older adults in rural areas: findings from a national dissemination. Int J Environ Res Public Health.

[17] Arksey H, O'Malley L (2005). Scoping studies: towards a methodological framework. Inter J Soc Res Method.

[18] Tricco AC, Lillie E, Zarin W, O'Brien KK, Colquhoun H, Levac D, Moher D, Peters MDJ, Horsley T, Weeks L, Hempel S, Akl EA, Chang C, McGowan J, Stewart L, Hartling L, Aldcroft A, Wilson MG, Garritty C, Lewin S, Godfrey CM, Macdonald MT, Langlois EV, Soares-Weiser K, Moriarty J, Clifford T, Tunçalp Özge, Straus SE (2018). PRISMA extension for scoping reviews (PRISMA-ScR): checklist and explanation. Ann Intern Med.

[19] Zecevic AA, Salmoni AW, Speechley M, Vandervoort AA (2006). Defining a fall and reasons for falling: comparisons among the views of seniors, health care providers, and the research literature. Gerontologist.

[20] Covidence.

[21] Braun V, Clarke V (2023). Toward good practice in thematic analysis: avoiding common problems and be(com)ing a researcher. Int J Transgend Health.

[22] Roberts K, Dowell A, Nie J (2019). Attempting rigour and replicability in thematic analysis of qualitative research data; a case study of codebook development. BMC Med Res Methodol.

[23] Meucci RD, Runzer-Colmenares FM, Parodi JF, de Mola CL (2020). Falls among the elderly in Peruvian Andean communities and the rural far south of Brazil: prevalence and associated factors. J Community Health.

[24] Johnson S, Jeffery B, Bacsu J, Abonyi S, Novik N (2016). Voices of senior rural men and women on falls and fall-related injuries: “If I fall outside and get hurt, what would I do?”. Educ Gerontol.

[25] Ong MF, Soh KL, Saimon R, Saidi HI, Tiong IK, Myint WW, Mortell M, Japar S (2024). Predictors of fall protection motivation among older adults in rural communities in a middle-income country: a cross-sectional study using the Protection Motivation Theory. J Adv Nurs.

[26] Santos FD, Lange C, Llano PMPD, Lemões Marcos Aurélio Matos, Pastore CA, Paskulin LMG, Costa AEKD, Raymundo JLP (2019). Falls of elderly people living in rural areas: prevalence and associated factors. Rev Bras Enferm.

[27] Tang HT, Vu HM, Tang HT, Tran PT, Tran LV, Nguyen CD, Nguyen TQ, Nguyen CMT, Tran KQ, Luong HX (2023). Knowledge, attitude and practice on fall risk factors and prevention among rural older community-dwellers in Vietnam. PLoS One.

[28] Gardiner S, Glogowska M, Stoddart C, Pendlebury S, Lasserson D, Jackson D (2017). Older people's experiences of falling and perceived risk of falls in the community: a narrative synthesis of qualitative research. Int J Older People Nurs.

