# Supplementary figures and images for: Falls Prevention Among Older Adults in Rural Communities: Protocol for a Scoping Review
Source: JMIR Res Protoc. 2025 Jul 21;14:e63716. doi: 10.2196/63716 (PMC12322609; doi:10.2196/63716)

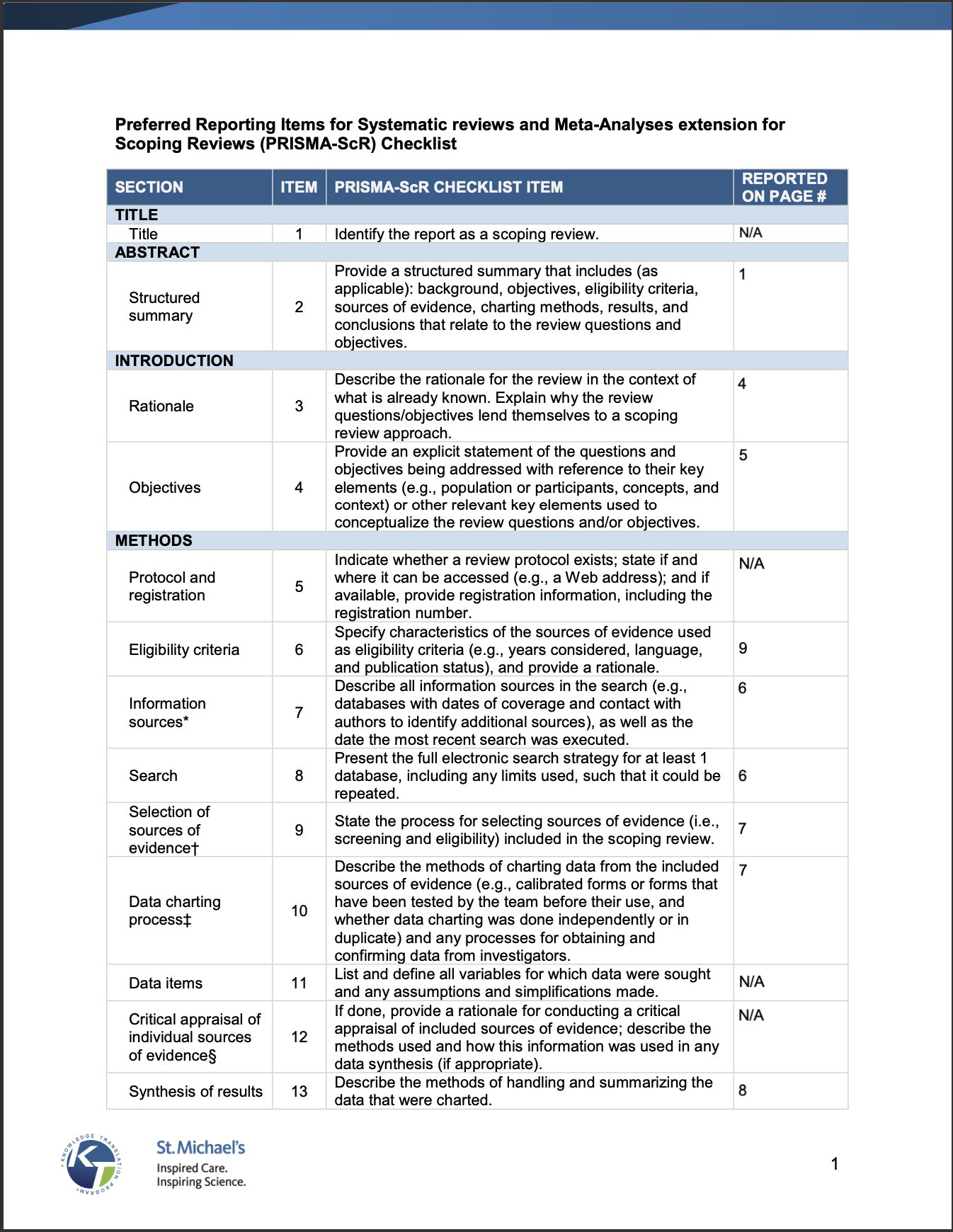


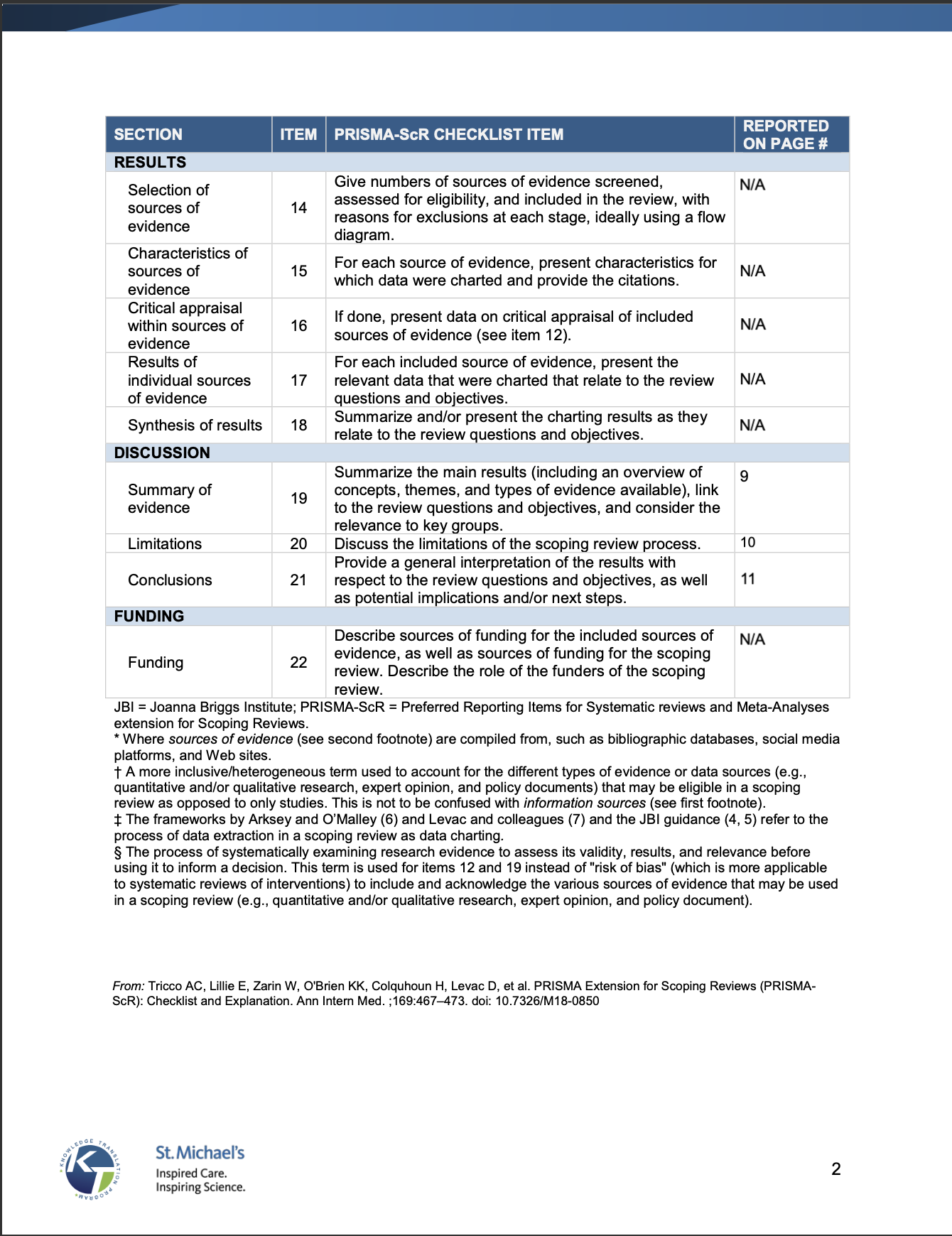

Supplement: Multimedia Appendix 1 [file resprot_v14i1e63716_app1.docx]
